# The History and the Future of the Psychology of Filial Piety: Chinese Norms to Contextualized Personality Construct

**DOI:** 10.3389/fpsyg.2019.00100

**Published:** 2019-01-30

**Authors:** Olwen Bedford, Kuang-Hui Yeh

**Affiliations:** ^1^Department of Psychology, National Taiwan University, Taipei, Taiwan; ^2^Institute of Ethnology, Academia Sinica, Taipei, Taiwan

**Keywords:** filial piety, filiality, indigenous psychology, Chinese culture, intergenerational relations, morality, elder care familism

## Abstract

In the field of psychology, *filial piety* is usually defined in terms of traditional Chinese culture-specific family traditions. The problem with this approach is that it tends to emphasize identification of behavioral rules or norms, which limits its potential for application in other cultural contexts. Due to the global trend of population aging, governments are searching for solutions to the accompanying financial burden so greater attention is being focused on the issue of elder care and its relevance to filial practices. We contend that the psychological investigation of filial piety in Chinese societies has progressed to the point that it can now provide a solid structure for research targeting intergenerational relations in other cultures. We describe an indigenous psychology approach that integrated Chinese historical, philosophical, and social trends to construct a model of filial piety in terms of the dual reciprocal and authoritarian filial aspects underlying parent–child relations: the dual filial piety model (DFPM). We use this model to re-conceptualize filial piety from its usual definition as a set of Chinese culture-specific norms to a contextualized personality construct represented by a pair of culturally-sensitive psychological schemas of parent–child interaction. We then describe how the DFPM can provide a framework for research on filial relations on individual, structural, societal, and cross-cultural levels. We conclude with a discussion of how the model may be able to integrate and extend Western research on intergenerational relations and contribute to the issue of elder care beyond Chinese societies.

## Introduction

Filial piety (*xiao*) is the core pillar of Confucian ethics (Ho, 1986). It specifies moral norms that encompass material and emotional aspects of the parent–child relationship. The character *xiao* 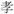 is comprised of an upper component representing *age* and a lower component representing *child*, indicating that the child supports and succeeds the parent. Filial piety not only specifies norms within the family, it also provides the social and ethical foundations for maintaining social order, and thus a stable society. It has provided the moral underpinning for Chinese patterns of parent–child relations and socialization for millennia.

Research interest in parent–child relations is on the rise due to the global trend of population aging and the growing issue of elder care (e.g., Glass et al., 2013; North and Fiske, 2015). Researchers around the world are investigating the influence of filial norms on adult children’s support of their elderly parents in order to share the public financial burden of elder care with the family system (e.g., Lowenstein and Daatland, 2006; Gans et al., 2009). Whereas Chinese filial norms encompass reciprocal expectations for parent–child relations, as well as social structure, ethical requirements, and power dynamics (Ikels, 2004), scholars in Western societies tend to define filial norms in a much more limited way. They portray them in terms of a generalized one-way expectation regarding adult children’s responsibility to support their aging parents (Silverstein et al., 2006), and conceptualize them as separate from the religious and social contexts, which are seen as situational influences on individual filial beliefs (Gans et al., 2009).

The question that arises is: can a seemingly culture-bound concept such as Chinese filial piety provide insight applicable to parent–child relations in other cultural contexts? Given the centrality of filial piety to Chinese culture, it is a highly developed construct. It is plausible that insights gained from studying parent–child relations in Chinese culture may shed light on aspects of parent–child relations that are less evident in other cultures, just as sociologist Goffman (1959) relied on the Chinese conceptualization of face in constructing his famous universal dramaturgical theory of face.

In this paper, we contend that the psychological investigation of parent–child relations in Chinese societies has progressed to the point that it can now provide a solid structure for research targeting intergenerational relations in other cultures. Specifically, we propose that filial piety is a type of contextualized personality construct, and as such it can provide a platform for research on parent–child relations in any culture.

In order to demonstrate this claim, we first review the foundations of filial piety in Chinese culture and describe the development of the modern psychology of filial piety through research conducted in Chinese societies. We highlight development of the dual filial piety model (DFPM) (Yeh, 2003; Yeh and Bedford, 2003) based on a Chinese indigenous psychology approach. Using this model, we re-conceptualize filial piety from its usual definition as a set of Chinese culture-specific norms to a contextualized personality construct representing the underlying psychological mechanisms in the parent–child relationship. With this perspective, the DFPM can provide a framework for research on intergenerational relational processes and schemas on individual, structural, societal, and cross-cultural levels. In the final section, we indicate how the re-conceptualized model can integrate and extend Western research on parent–child relations.

## The Philosophy of Filial Piety

The foundation of filial piety lies in ancestor worship (Hsu, 1975). Emperors traced their line of descent from Shang Di, the great founder ancestor god. If the ancestors received the appropriate sacrifices they could provide guidance on important governmental decisions and protect the dynasty (Hsu, 1975). The duty to provide for the ancestors encompassed the emperor and common people alike. The head of a family ruled over his relatives as the emperor ruled over his subjects. Thus, ancestor worship not only provided the organizing principle for the kinship system, but also for role relations in Chinese society in general.

Confucius (551–479 BCE) refined the filial obligations of ancestor worship (Wei, 1969). He lived at a time of great chaos and feudal bickering, so he condensed prevailing beliefs into a practical philosophy, the Way of Humanity, to ensure harmony in the family and thus society. Instead of emphasizing communication with ancestors, he emphasized family, virtue, and orderly social relations (Bi and D’Agostino, 2004). Confucius’ codified works became the cornerstone of education, and the guidelines for moral conduct. They still provide the template for Chinese social structure (Hwang, 1987).

Confucius’ Way of Humanity specifies that two ethical principles should guide social interaction: favoring the intimate (giving preference to those with whom one is closest) and respecting the superior (giving deference to those who are older) (Hwang, 1987). Each is described in the following.

### Favoring the Intimate: Pre-Chin Era (521 to 221 BCE)

During Confucius’ time, filial piety emphasized the ethical principle of favoring the intimate, which ensures preferential treatment of one’s kin (Hwang, 1987). Confucius described parent–child interaction as motivated by natural affection and the principle of bao (reciprocity), which requires that all helpful behavior be returned (Hsu, 1975). Affection and bao should flow both ways. However, because children have a fundamental obligation to their parents for giving them life, their obligation can never be fully repaid. Thus, motivated by affection, children can return the care they received from their parents by carrying out filial duties such as being respectful and looking after their parents in their old age (Yeh, 2003).

### Respecting the Superior: Han to the Qing Dynasty (206 BCE to 1911 AD)

The motivation underlying the practice of filial piety shifted from the principle of favoring the intimate to the principle of respecting the superior during the Han dynasty (206 BCE–220 AD), and retained this emphasis through the end of the Qing dynasty (1911) (Hsu, 1975). This principle specifies that the person in the superior position should play the role of the decision-maker to ensure family solidarity and prosperity (Hwang, 1987). Accordingly, it is ethically proper for the person in the superior position to make decisions concerning those in inferior positions.

Under this principle, the practice of filial piety required submission to hierarchical authority and suppression of self-autonomy. Children discounted self-needs to achieve parental desires (Yeh, 2003). This principle justified not only absolute parental authority over children but also, by extension, the authority of any person of an elder generation over those who are junior (Hwang, 2012). The shift in emphasis to the principle of respecting the superior was related to the need to strengthen political sovereignty, with patriarchal parental authority as a representation of the emperor’s absolute authority (Miao, 2015).

### Modern Filial Piety

The majority of elderly people living in Chinese communities during the 19^*th*^ and early 20^*th*^ centuries lived with their married child, usually their son (Ikels, 2004). Their son, daughter-in-law, and grandchildren provided them with physical, emotional, and financial support. Daughters provided their own parents such support only until they married. After marriage, their primary responsibility shifted to their husbands’ parents (Whyte, 2004). As in imperial times, obedience and submission motivated filial piety (Yeh, 2003).

After WWII, Chinese societies began to go through political reform, marketization, and social change as people adapted their lifestyles and value systems. These developments gave rise to questions about the impact of those changes on the patterns and structures of social relationships (Yang et al., 1989). Chinese scholars noticed the trend toward smaller families, greater geographic mobility, and the expansion of women in the workplace. They began to wonder about the harmful effects of filial beliefs in the new modern context (Yeh, 1999). For example, filial piety seemed to inhibit the individual’s independence, suppress creativity, and eliminate personal desires and interests (e.g., Liu and Lin, 1988). They also expressed concern that exposure to the Western ideologies of freedom and independence was giving rise to internal conflict between being filial according to traditional standards, and being self-responsive, independent, and modern (e.g., Ho, 1996).

A number of researchers indicated that filial beliefs may be waning with industrialization and modernization (Yeh, 1997). Some focused on the rapid economic development in Taiwan and argued that Western influences were diminishing the relevance of traditional filial obligations (e.g., Yang, 1988). Others focused on the political climate in the PRC (e.g., Lu, 1990). For example, the Socialist Transformation Movement and the Cultural Revolution led some scholars to doubt the survival of filial piety under Chinese communism, especially since PRC leaders criticized traditional values as feudal remnants to be eliminated (Whyte, 2004). To focus loyalty on the central government and Communist ideology and away from ancestors and parents, China’s authorities created civil rights focused on the individual rather than the family. They passed legislation specifying that daughters and sons share equal responsibility for their birth parents (Miao, 2015).

## Psychological Study of Filial Piety in Chinese Societies

Filial piety has long been an important topic to philosophers and historians. Psychologists in Chinese societies only began systematic study of filial piety in the past few decades. Personality and social psychologists wanted to better understand how the process of societal modernization impacted the content and structure of people’s psychological make-up (Yang et al., 1990). They could not ground their research in classical modernization theory (as Western scholars had) because it equates modernization with Westernization. Consequently, many avoided the ongoing debates surrounding sociological theories of societal modernity and instead adopted a social psychological approach (Yang, 2003).

### Dispositional Approaches

Initially, psychology researchers adopted a psychometric dispositional approach that entailed applying Western methodologies and instruments to local participants (Hwang, 2012). This approach uses the cultural context as a predictor in Western psychology models. Researchers defined filial piety in terms of traditional Chinese norms, and focused on finding ways to measure the strength of an individual’s filial beliefs or attitudes. They then related these scales to other psychological traits or behaviors. The result was two opposing sets of findings that generated debate as to whether filial piety had an overall harmful or helpful impact on individual psychological development and interpersonal relationships, as well as conflicting findings on the question of whether filial piety was waning in influence (see Yeh, 2003 for a review of harmful and beneficial perspectives).

For example, David Ho was one of the first to develop a filial piety measure (Ho and Yu, 1974). He identified filial attitudes that correlated with traditional parental attitudes such as control, protection, harshness, and neglect, as well as inhibition of self-expression, independence, and creativity (Ho, 1987). Ho (1994) found that filial attitudes were most prevalent among those of low education and socio-economic status, and so concluded that filial beliefs may be diminishing in modern societies. This approach equates filial piety with Chinese cultural traditions and focuses on the decline of traditional norms supporting filial piety.

In contrast, researchers with a more relational focus of investigation found that filial piety supports warmth, love, harmony, and close family ties, and thus has a beneficial effect on personal growth and interpersonal relationships (e.g., Yang, 1988; Ishii-Kuntz, 1997). Their studies demonstrated that filial values were not waning, and that the mutual interdependence of family members remained strong despite changes in living arrangements and income opportunities for women (see Yeh, 2003 for a review of related studies).

Some scholars have criticized dispositional studies as “explorative, and not very profound or systematic” (Yang et al., 1990, p. 66), and as “Americanized in the sense that nearly all uncritically borrow theories, concepts, methods, and tools developed…for Euro-American subjects” (Yang, 2006, p. 285). Researchers applying this approach were unable to tap into changes in the nature of contemporary filial piety; they could only indicate the extent of respondents’ recognition of traditional filial beliefs. In other words, they did not investigate the mechanisms or processes underlying filial piety (the deep structure of human functioning), but focused on the surface content of traditional beliefs. Moreover, since they investigated different aspects of filial belief instead of the complete concept, the findings they reported conflicted with one another (Yeh, 2003).

### Moral Development Approaches

Filial piety is the central pillar of the Confucian ethical system (Yeh and Bedford, 2019). Children in Chinese societies are taught that the way they treat their elders is a measure of their moral worth. Thus, some psychology researchers focused on understanding the cognitive stages of filial piety development from the perspective of moral development. Most applied Kohlberg’s stages using dilemma scenarios, which was the dominant paradigm for research on individual moral development at the time (e.g., Snarey, 1985; Ma, 1988). Kohlberg (1981) asserted that moral development progresses through differentiated hierarchical stages with a specified sequence, similar to Piaget (1972/1981) theory of cognitive development.

Filial piety research following Kohlberg’s paradigm encountered a different type of challenge. Kohlberg (1981) asserted that his stages of moral development were invariant for all people. However, he also defined the moral maturation representing the higher stages in terms of the Western ideology of rationalism, individualism, and liberalism. Chinese researchers who adopted Kohlberg’s approach did not initially question this assertion. They did not consider whether a theory developed in the context of Western moral values would applicable in a culture centered on Confucian ethics (Yeh and Bedford, 2019).

A number of the studies that applied Kohlberg’s paradigm to collect moral reasoning data in Chinese societies encountered the problem that many participants’ responses could not be scored with Kohlberg’s standardized system (Snarey, 1985). Most unscorable responses were related to filial piety (e.g., Ma, 1997), indicating that filial piety may have implications for moral decision-making. Doubts about the universal applicability of Kohlberg’s stages were strengthened when reviews of cross-cultural studies of Kohlberg’s stages concluded that the higher stages are not universal because they fail to take into account non-Western philosophies (e.g., Edwards, 1981).

### The Indigenous Psychology Approach: The Dual Filial Piety Model

To overcome the conflicting results and cultural mismatch encountered in prior filial piety studies, some researchers in Chinese societies began to adopt an indigenous psychology approach (e.g., Yang et al., 1990; Yeh, 1997, 1999). This approach purposefully incorporates a cultural perspective into both conceptual development and theoretical construction. It entails creation and application of theories, concepts, methods, and tools that represent local structures and processes (Yang, 2006). In the following, we describe in some detail the indigenous model developed to represent the psychological mechanisms supporting Chinese filial piety.

The review presented in the philosophy section suggests that varied aspects of filial piety were highlighted in different stages of China’s development. The filial concept during the pre-Chin Era (before 221 BCE) focused on the reciprocal affection of the parent–child dyad. The filial concept during the period from the Han to the Qing dynasties (202 BCE-1911 AD) emphasized the family role hierarchy (Hamilton, 1990).

Yeh (2003) integrated these findings from Chinese history and philosophy to construct a dual-factor model of filial piety. Each factor has psychological meaning at the individual level, and also reflects the influence of historical, societal, and cultural contextual factors. The DFPM is comprised of two higher-order factors that correspond to the two focal filial piety attributes: reciprocity and authoritarianism. Yeh and Bedford (2003) provided empirical evidence for the contents of each type of filial piety and validated a dual filial piety scale. Each factor is described in the following.

Reciprocal filial piety (RFP) develops out of genuine affection from long-term positive interaction with one’s parents in daily life. It is rooted in intimacy and the quality of the parent–child relationship. It encompasses emotionally and spiritually caring for one’s parents out of authentic gratitude for their effort and sacrifice, as well as physical and financial care for one’s parents as they age. RFP fulfills the psychological need for relatedness between two individuals within the context of the parent–child relationship (but not the family role dyad). It generally manifests in terms of children’s voluntary support behaviors as expressions of love and care for their parents.

Reciprocal filial piety tends to be positively associated with a higher level of education and a higher socio-economic status. Women tend to score higher on RFP than men. It develops through intergenerational communication and sharing in daily life, so it is positively correlated with interpersonal skills (e.g., self-disclosure and empathy), better psychosocial adjustment, and emotional support of parents (Yeh et al., 2009). RFP also positively correlates with life satisfaction (Wong et al., 2010), and mediates the influence of supportive parenting on young adults’ happiness (Chen et al., 2016).

Authoritarian filial piety (AFP) is guided by obedience to role obligations based on the family hierarchy. It entails suppressing one’s own wishes to comply with one’s parents’ wishes (because of their seniority). Continuing the family lineage and maintaining the family reputation are important. Parents are role models who represent absolute authority during their children’s development and socialization. AFP develops through children’s normative reactions to satisfying parental demands or expectations. AFP fulfills the need for social belonging and collective identity.

Authoritarian filial piety beliefs are positively associated with less education and lower socio-economic status. Men tend to score higher than women in AFP, and it correlates positively with traditional conservative attitudes (e.g., male superiority and submission to authority) and maladaptation (e.g., neurotic personality traits, depression and anxiety) (Yeh, 2006). Because AFP often involves self-suppression, it is more likely to correlate with personal stress than RFP (Yeh, 2006), and has a only a low correlation with emotional support of parents (Yeh, 2009).

Reciprocity and authoritarianism form two intertwined aspects of Chinese filial piety grounded in historical development of the concept. They are not mutually exclusive, but coexist within an individual. They may simultaneously function to varying degrees that depend upon the circumstance (Yeh and Bedford, 2004). They may also both promote the same outcome. For instance, both reduce parent–adolescent conflict at the family level, although the effect of RFP (via reconciliation) is generally more significant than that of AFP (via inhibition). Both also promote intergenerational support: RFP via accumulated affection, and AFP by regulating behavior so that the minimum social expectations for the role of the child are met. The detailed psychological features of the two filial dimensions are summarized in Table 1.

**Table 1:** The dual filial piety model: psychological schemas for interaction with parents.

	Reciprocal filial dimension	Authoritarian filial dimension
Psychological needs and manifestations in different development stages	Need for interpersonal relatedness (toward another individual)	Need for collective identity (toward society or generalized others)
	**Infancy to adolescence**: Create emotional safety and affective bonding with parents (main caregiver) through expression of love or affection **Adulthood**: Continuously strengthen affection and bonding with parents; understand and support parents’ life needs	**Infancy to adolescence**: Avoid punishment and gain social reward (e.g., parental praise) through learning to obey parental demands **Adulthood**: Practice the social role of child according to common behavioral standards
Features of psychological functioning	Simultaneously satisfy the mutual needs (for relatedness and emotional safety) of parent and child	Consider others’ needs (parents, spouse, or the whole family) prior to personal needs
Structural features inherent in the parent–child relationship	Equal status between two individuals; Need fulfillment is based on individual traits or differences	Unequal status between the different roles within the family hierarchy; Need fulfillment is based on specific role norms
Confucian ethical principles	Principle of favoring the intimate	Principle of respecting the superior

*Adapted from Tsao and Yeh (2019).*

### Psychological Insights From the DFPM

The DFPM has led to at least three important insights. First, rather than designating people as filial or un-filial, the DFPM allows for a more nuanced examination of the psychological mechanisms underlying the affective reactions and social behavior associated with filial piety. It provides a more comprehensive framework for understanding the personal practice of filial piety (including its motivation and expression) based on the interaction between RFP and AFP. For example, the DFPM identifies four possible modes of personal interaction with parents (Yeh and Bedford, 2004).

People high in both reciprocal and authoritarian filial dimensions operate in a *balanced mode.* They are able to simultaneously consider personal choices and role obligations. They have a deep and intimate bond with their parents and easily find ways to combine their parents’ needs with their own. People high in RFP and low in AFP operate in a *reciprocal mode.* They have a positive relationship with their parents and good communication. They emphasize personal choices over role obligations, and experience filial piety as authentic love rather than self-sacrifice. People with low RFP and high AFP operate in an *authoritarian mode* and have a less intimate and more obedient relationship with their parents. Authoritarians focus on role obligations and perceive filial piety as self-suppression or self-sacrifice. They may find it difficult to satisfy their parents’ needs. People functioning in the *non-filial mode* are low in both RFP and AFP and isolate themselves from their parents. Non-filials have low identification with their family and child roles. Psychological mechanisms other than the dual aspects of filial piety, such as egocentrism, may guide their behavior toward their parents.

Second, the DFPM can explain previous divergent findings on the impact of filial piety in Chinese societies. As noted, early research resulted in conflicting findings as to whether filial piety had an overall harmful or helpful impact in the context of modern society. Some researchers cautioned against its negative impact such as inhibition of self-expression and independence (e.g., Ho, 1987). Others found that filial piety supports warmth and harmony, with a beneficial effect on personal growth and interpersonal relationships (e.g., Ishii-Kuntz, 1997). With the DFPM, it is clear that these two sets of findings do not conflict. Instead, they represent the two coexisting dynamic fundamental aspects of filial piety that must be considered together in order to have a complete understanding of the role of filial piety in modern Chinese societies.

Third, scholars have noted an overlap between Chinese filial piety and values or attitudes found in other cultures. The DFPM highlights the necessity of considering both aspects of filial piety in order to gain a complete picture of its role in Chinese societies. Similarly, it is also necessary to consider both aspects of Chinese filial piety when comparing it to related filial concepts in other societies. However, in many such comparisons, researchers have used incomplete concepts to represent Chinese filial piety, meaning that they did not make a comprehensive evaluation.

For example, Schwartz et al. (2010) observed that although Hispanic familism and Chinese filial piety originate from different parts of the world, they share common elements in that they stress social ties over individual desires. In other words, they share transcultural dimensions. Schwartz et al. (2010) demonstrated that both familism and filial piety group together under a single factor of family/relationship primacy, which is equivalent across gender and ethnicity. However, the items Schwartz et al. (2010) used to measure the concept of filial piety all focused solely on authoritarian aspects (AFP), and no items addressed reciprocal aspects (RFP). Unger et al. (2002) also found overlap between Hispanic familism and filial piety. Unger et al. (2002) definition of filial piety relied solely on the work of Ho (1994), who detailed only AFP characteristics. Thus, it appears that at least in terms of social belongingness schema, some characteristics of Hispanic familism are similar to AFP, but the comparison is incomplete since RFP aspects of Chinese filial piety were not considered. This observation also invites the question of whether reciprocal aspects of Hispanic familism exist.

Another example comes from scholars who have used terms like *filial duties* and *filial obligations* to describe the nature of North American parent–child relations. Blustein (1977) proposed that because Western parent–child relations are based on affection and intimacy, there are inherent limits on the nature of the obligations they entail. In Blustein’s view, filial duties are based on gratitude and not indebtedness, and so cannot be expressed in terms of repayment, but only through an ongoing relationship that affirms the special importance of the parent. English (1979, p. 147) directly answered the question “what do grown children owe their parents?” with a single word: “nothing.” She contended that the parent–child relationship is best characterized as an ongoing friendship with spontaneous affection, and that love is the correct ground of filial obligation. Dixon (1995) likewise argued that the voluntary and loving nature of the relationship is the most robust basis of children’s filial behavior. All three of these scholars provided reasoned arguments about the moral foundations of parent–child obligations, rights, and duties to one another. Although the nature of these arguments reflects reasoning that is nothing like Confucian ethics, the foundation of filial relations in gratitude and spontaneous affection and love that they describe as the ideal for parent–child relations in the West is identical to that supported by the reciprocal aspect of filial piety in Chinese societies. The manifestation or expression of affection may differ by culture, but affection-based interaction between children and parents exists in all cultures.

Approaches to Chinese filial piety that focus on traditional norms tend to emphasize aspects related to AFP (as in the direct cultural comparison with Hispanic familism). That approach does not allow the cultural similarities in RFP-related norms to be considered. As can be seen from these examples, exploring parent–child relations in terms of the dual aspects of the DFPM may help to illuminate cultural similarities as well as differences in approaches to parent–child relations.

### From Indigenous Theory to Cultural Psychology

Researchers initiated indigenous psychology in Chinese societies to overcome shortcomings in the application of Western theory to the local context and to provide actionable results to solve local problems. However, the ultimate academic mission of indigenous psychology is to contribute to development of formal theories with relevance to various cultures (Hwang, 2005). This perspective corresponds to the viewpoint of cultural psychology.

According to Shweder et al. (2006) “one mind, many mentalities” argument, cultural psychology has a psychological side and a cultural side. The psychological side examines how individual persons think and act in light of their particular goals, values, and understandings of the world. This side probes the universal part of the human mind. The cultural side entails examination of socially-assisted processes of learning and schema activation that are associated with becoming a member of a particular group. This side investigates the diverse part of human mentalities. In other words, cultural psychology entails reciprocal investigation of the psychological foundations of culture, and the cultural foundations of mind. They are interdependent; culture and psyche make each other up. Context and meaning are theoretically represented as part of the psychological system, and not as external influences or factors.

Our task here is to take the DFPM, developed in a Chinese society from analysis of Chinese historical and philosophical traditions, and bring it into the realm of cultural psychology where it can represent both the deep structure of human functioning to investigate aspects of the universal mind, as well as the surface content of culture reflecting diverse mentalities. In the following, we explain how the notion of filial piety can be re-conceptualized so that the DFPM can be applied more broadly using the approach of cultural psychology.

## Examining Parent–Child Relations as a Contextualized Personality Construct

In our review of the development of the psychology of filial piety, we described a shift in use of the term *filial piety*. Initially, the term was mostly used to refer to a set of traditional Confucian norms. Cognitive psychologists then investigated it as a series of stages of Confucian moral development. Use of an indigenous psychology approach allowed filial piety to be construed in terms of the dual motivations and processes underlying parent–child relations over the course of a person’s entire lifespan. With this final definition, we propose that filial piety is best characterized as a *contextualized personality construct*.

*Contextualized personality* refers to “stable patterns of thought, feelings, and behaviors that occur repeatedly within a given context” (Heller et al., 2007, p. 1229). It captures the idea that personality manifests in different ways across various social roles and contexts. Personality is not merely a collection of traits. Personality emerges from the interaction of the individual with the environment, and is expressed in terms of goals and motivations in addition to traits (Dunlop, 2015). These goals or motivations correspond to particular interpersonal relationships or sociocultural contexts. They are person-in-context variables—personality characteristics that are inseparable from the context in which they are pursued (Nasby and Read, 1997). In other words, a contextualized personality construct connects individual-level motivations or goals to their social context.

Contextualized personality researchers highlight social roles as ideal for contextualizing personality motivations. Social roles can be organized around the two universal dimensions of affiliation and power (Roberts, 2006), which allow individuals to meet the fundamental human needs of interpersonal relatedness and social belonging. The DFPM uses the parent–child social roles to contextualize these two needs: interpersonal relatedness (reciprocal filial piety) and social belonging (authoritarian filial piety).

A contextualized personality approach to filial piety focuses first on parent–child interaction as the core context for understanding underlying filial motivations. The inherent structure of the parent–child relationship provides the appropriate cues for integrating the various contents of filial norms into the underlying filial psychological mechanisms.

Because this approach focuses on the psychological mechanisms underlying parent–child relations (a universal context), and not on cultural content, it has the potential for application in any culture. From this perspective, the DFPM can provide a framework for four levels of analysis (see Table 2). It can be used to examine basic individual motives in the context of parent–child relations; the structural properties of the parent–child relationship; social changes in filial norms; and differences across groups or societies in the expression of individual needs, relational structure, and filial norms.

**Table 2:** Theoretical implications of the DFPM at different levels of analysis.

Level of analysis	Corresponding implications
Basic psychological needs (of children)	RFP: interpersonal relatedness AFP: social belonging and collective identity
Structural properties of parent–child relationship	RFP: horizontal relationship between two individuals AFP: vertical relationship based on family role hierarchy
Social change	RFP: core aspect of filial relations (relatively free from the impact of social change) AFP: changing aspect of filial relations
Cross-cultural comparison	RFP: psychological prototype of filial relations (deep structure) AFP: cultural prototype of filial relations (surface structure)

*RFP, reciprocal filial piety; AFP, authoritarian filial piety. Adapted from Tsao and Yeh (2019).*

### Individual Level

As a contextualized personality construct, filial piety develops from childhood and has enduring influence on the parent–child relationship. The DFPM identifies two basic universal psychological motives at the individual level: the need for interpersonal relatedness (RFP) and the need for social belonging and collective identity (AFP). Interpersonal relatedness needs correspond to beliefs about the parent–child interpersonal connection as two individuals (not in terms of their social roles). The need for social belonging and for collective identity corresponds to beliefs about adherence to social norms and fitting in to ensure membership within a larger group.

We provided psychological details pertaining to each type of filial need (as manifest in Chinese societies) in the previous section, so here we provide an example related to elder care that demonstrates the difference in outcome that may occur when individuals interpret the same behavior as motivated by affection as opposed to being motivated by a desire to comply with social norms. A recent study on end-of-life caregiving showed that the reciprocal filial dimension not only correlated with the caregivers’ (adult children’s) reduced burden and stress, but also with the elderly parents’ enhanced self-worth (Chan et al., 2012). RFP has also been shown to correspond to adult children’s intention to remain as caregivers of their parents and the quality of their care (Hsiao and Chiou, 2011). Care attributed to the authoritarian filial dimension was not associated with these positive benefits in either of these studies.

### Structural Analysis

The DFPM captures the horizontal–vertical duality of the parent–child relationship. The reciprocal dimension encapsulates the horizontal aspect of the parent–child dyad. It corresponds to an equal relationship between two particular individuals who can understand each other only through interaction and communication. The authoritarian dimension captures the vertical aspect and relates to the hierarchical relationship between two family roles (parents and child) for which there exist some universal elements (such as the dependence of the child on the parent), and some cultural elements (such as the priority on the parent’s rights in Chinese culture).

This vertical–horizontal duality of the parent–child relationship can reflect meaningful individual differences in interaction patterns with parents. It is like a personality characteristic that develops naturally in response to the first interpersonal context everyone encounters after birth. We note that these two aspects of parent–child interaction are often entwined in daily life. We distinguish them at a theoretical level in order to parse the possible patterns of parent–child interaction.

This vertical–horizontal duality has implications at both individual and societal levels. China provides an interesting case example. China’s imperial rulers emphasized authoritarian moralism and deference (AFP) to consolidate their power. Communist leaders initially tried to eradicate these Confucian beliefs to ensure the centrality of the state (An, 2009). More recently, China’s authorities have switched tactics to focus on reciprocal aspects of filial piety in order to address their looming elder-care crisis due to population aging (Xu, 2001). Rather than emphasizing obedience, they now focus on support for aging parents. Because there are no state welfare services, adult children, especially daughters, are being urged to support their elderly parents. The 1980 Marriage Law even gave parents who cannot provide for themselves the right to demand payments from their children (Qi, 2015). In the mid-1980s, China established the Family Support Agreement (FSA), a voluntary contract between older parents and adult children concerning parental provisions. Although the FSA’s moral persuasion is based on filial ethics, violations are subject to legal prosecution (Chou, 2011). Local governments have also enacted provisions such as requiring all adult children to pay their elderly parents a monthly allowance and take them for a monthly haircut.

In effect, China’s authorities are relying on authoritarian (vertical) means to try to enhance reciprocal (horizontal) filial relations. According to the DFPM, this approach is not likely to be effective, and the evidence bears this supposition out. A study on the FSA found that it erodes spontaneity, flexibility, and affection in the practice of filial piety (elements that correspond to RFP), and thus harms relationships and results in more family lawsuits (Chou, 2011).

### Social Change Analysis

The DFPM is able to capture changes over time in the endorsement of filial norms within societies, and differences in filial norms across societies. The argument supporting these two levels of analysis is similar. Both the reciprocal and authoritarian dimensions have a psychological basis. But, for the purpose of comparison over time or across cultures, they each correspond to a different prototype.

Reciprocal filial piety represents the psychological prototype of filial relations in the sense that it pertains to universal psychological processes relevant to the parent–child context. The psychological functions of filial piety at the individual level connect to the child’s personal motives for filial practices, and represent the deep structure of the filial mind. RFP is thus likely to remain stable over time and to share characteristics across cultures (which we address in the following section).

Authoritarian filial piety represents the social/cultural prototype of parent–child relations through the parents’ role as a personalized representation of the social/collective authority. Even though the absolute authority of parents often gradually weakens as children grow into adolescence and adulthood, AFP may still reflect internalized role obligations contained in cultural filial norms. Thus, AFP reflects the schema for social belongingness and collective identity associated with becoming a member of a particular group. Accordingly, over time it may evolve with broader social changes. It may also capture meaningful differences across cultures in the surface content of filial norms and beliefs. In other words, AFP is most relevant to understanding the impact of the changing social context on, or cultural differences in, filial relations.

An example of social change analysis relates to an early insight from application of the DFPM in Chinese societies. Early studies conducted prior to development of the DFPM measured the extent of endorsement of traditional filial norms (such as obedience and deference, which are related to AFP), and thus reported a decline in relevance of filial piety (e.g., Ho and Yu, 1974; Ho, 1996). These studies accurately identified that changing social conditions were connected to changing levels of endorsement of some traditional filial values, but the researchers erred in assuming that the surface content of the traditional norms (related to AFP) represented the whole of filial piety. In fact, studies conducted around the same time that focused solely on intergenerational caring and support (aspects of RFP) did not report a change in endorsement of traditional filial beliefs (e.g., Yeh, 1997; Yue and Ng, 1999).

Another example comes from a study of Chinese immigrant families in New Zealand. The authors acknowledged that authoritarian aspects of filial relationships may change due to the new context, but noted that relational aspects of filial belief remained consistent (Liu et al., 2000). Recent studies using the DFPM in Chinese societies likewise support the contention that endorsement of authoritarian aspects of filial piety is diminishing (Chow, 2006; Chan et al., 2012). However, these findings do not mean that AFP beliefs are no longer relevant. For example, a 2-year longitudinal study of Chinese adolescents concluded that although participants had a higher level of reciprocal than AFP, AFP still had relevance for understanding parent–child interaction, particularly obedience (Liu, 2013).

### Cross-Cultural Applications

In their article conceptualizing psychological universals, Norenzayan and Heine (2005) stated that a psychological phenomenon qualifies as a functional universal if the shape of the relationship between the variables is the same, even if the strength of the pattern differs across cultures. Filial piety meets the requirements of a functional universal when it is conceptualized in terms of a contextualized personality construct. The DFPM encompasses the two universal psychological needs of interpersonal relatedness and social belonging in the universal context of the parent–child relationship. The model provides the structure to link these two motivations to the surface content of filial norms at the collective level, which may vary by culture. As a functional universal, the DFPM can be compared with concepts depicting intergenerational relations in other cultures. In particular, any concepts specifying children’s psychological processes in relating to their parents (such as their beliefs, attitudes, or perceptions of their parents) may correspond to one or both of the dimensions of the DFPM.

In this section, we review some western concepts of intergenerational relations, and suggest how the DFPM may integrate or extend them. This exercise demonstrates the cross-cultural applicability of the DFPM, and highlights multiple directions for future research on intergenerational relations in Western societies.

Few researchers have considered comparing filial piety and attachment style. Attachment style (Ainsworth and Bell, 1970) fits the conceptual requirements of a functional universal (Norenzayan and Heine, 2005). It can also be seen as a type of contextualized personality concept reflecting the schema of children’s interaction with their parents, so it can be directly compared with the DFPM. In the following, we compare attachment style and the DFPM from three aspects: (1) the mode of interaction with parents, (2) the critical period of development, and (3) children’s developmental task in their relationship with their parents.

First, the two frameworks classify interaction modes similarly. Secure attachment style corresponds to the balanced mode of filial operation (high RFP and high AFP). The avoidant attachment style corresponds to the non-filial mode. The ambivalent attachment style could correspond to both the reciprocal and authoritarian modes in that both types of children lack the capacity to balance personalized filial practices based on affection for the parent (RFP) with the social expectations for the role of children (AFP). Thus, children with the reciprocal mode of intergenerational interaction are more likely to be anxious about others’ doubts and criticisms of their personalized filial behaviors. Those with the authoritarian mode are more likely to feel stress due to parents’ extra demands and needs. Even though separation anxiety with parents is not the theoretical focus of the DFPM, the combination of RFP and AFP still covers a wider range of anxiety situations related to interaction with parents.

Second, according to attachment theory, the only task of a child is to form and maintain a strong bond with the parents until adulthood. In contrast, the DFPM elucidates the nature of the parent–child relationship across the lifespan, and encompasses both horizontal and vertical aspects. There is a major difference in the critical period of development. Attachment theory focuses on the specific interaction style originating in infancy; RFP focuses on the continuous accumulation of mutual understanding between parent and child across the lifespan, and the DFPM recognizes that the parent–child relationship helps children transition into adulthood and emphasizes the importance of a continued relationship with one’s parents after growing up.

Third, according to attachment theory, the major task of children is to strive for individual autonomy from the relationship with their parents (Ainsworth and Bowlby, 1991). In contrast, from the perspective of the DFPM, the major developmental task of children is to form self-volition by integrating numerous social roles, instead of just self-autonomy. The parent–child relationship becomes the basis for establishing other types of social relations or developing a more complex identity comprised of multiple social roles.

The overlap between attachment styles and filial modes indicates that the DFPM has the potential to incorporate well-known western conceptual counterparts. The differences suggest avenues to extend western intergenerational relations research. Although the influence of attachment style on a person’s subsequent interpersonal relationships has been broadly discussed in the literature, the concept of attachment has rarely been applied to investigate intergenerational interaction between adult children and their parents. Researchers have developed concepts other than attachment to describe intergenerational relations in later life. Some of these concepts regarding adult relationships with parents can also be integrated into the DFPM.

For example, the concept of *intergenerational ambivalence*, which refers to the experience of both positive and negative feelings toward parents (Luescher and Pillemer, 1998) can be expressed as an imbalance between RFP and AFP. The concept of (perceived) *parental authority* is used to predict adolescent life adaptation, social development, and delinquency (Smetana, 2000; Darling et al., 2008). It focuses on the vertical structure of the parent–child relationship as a counterpart to AFP. A third example involves an emerging concept, *moral capital* (Silverstein et al., 2012). It is defined as the stock of internalized social norms regarding children’s obligation to care for their older parents, and as a set of intergenerationally transmitted values that make up for the insufficiency of relying solely on children’s gratitude or emotional bonds with their parents. Researchers developed this concept to address with the issue of elder care in light of global population aging. Moral capital corresponds to AFP in representing role obligations based on the family hierarchy. It can guarantee children’s support of their parents even with a weak parent–child relationship.

These concepts that deal with parent–child relations after childhood are similar to AFP in their mechanisms, functions, and theoretical implications. However, Western researchers have not developed corresponding concepts similar to RFP. Findings from application of the DFPM in Chinese societies imply that this may be a fruitful area for future research with respect to elder care. For example, although previous studies in Chinese societies demonstrated beneficial effects of both the reciprocal and authoritarian filial dimensions on intergenerational support for elderly parents, RFP repeatedly corresponded with a stronger and broader effect. In Taiwan, RFP showed a significant positive relation with the frequency of financial support, household labor assistance, and emotional support toward elderly parents. In contrast, AFP only showed a positive relation with the frequency of household labor assistance (Yeh, 2009). Researchers reported similar results in China and Hong Kong using cross-national data from the East Asia Survey (Yeh et al., 2013). This consistent finding has had implications for government policy on elder care. Officials in Taiwan have started to shift away from population aging policies that focus on institutional care (which has not been well-accepted) and toward policies more focused on enhancing RFP over AFP. For example, the Taiwan Ministry of Education (2011) proposed highlighting RFP as the core family value in school curriculum, and the legislature inaugurated National Grandparents Day in 2010 with the goal of facilitating increased daily intergenerational interaction and mutual understanding.

## Conclusion

Filial piety has provided the moral underpinning for Chinese patterns of intergenerational relations and socialization for millennia. Psychologists in Chinese societies began to investigate filial piety systematically in the past few decades to better understand how the process of societal modernization impacted the content and structure of Chinese people’s psychological make-up. Now, researchers around the world are looking at filial beliefs and norms and parent–child relationships in effort to address the issues surrounding population aging and eldercare.

Early researchers in Chinese societies defined filial piety in terms of Chinese cultural traditions, limiting the implications of their findings to Chinese societies. Indigenous psychology researchers took a different approach. They integrated findings from Chinese history and philosophy into a psychological model of parent–child relations: the DFPM. Each factor in the model has psychological meaning at the individual level, and also reflects the influence of Chinese historical, societal, and cultural factors. From the perspective of cultural psychology, research that focuses on a universal context, such as the parent–child relationship, can result in insights that reflect the uniqueness of local characteristics as well as universal or shared features (Tsui, 2004).

In this paper, we proposed that filial piety as conceptualized in the DFPM is a functional universal (Norenzayan and Heine, 2005) contextualized personality construct (Dunlop, 2015). As such, it connects the two universal underlying individual filial motivations to the social role in which they are relevant. From this perspective, the DFPM balances universal human tenets, cultural diversity, and individual differences. It can thus support multiple levels of analysis of intergenerational relationships across life stages, both within and across cultures.

There is now a solid body of research applying the DFPM in Chinese societies. Findings from this research are being used to shape public policy and programs on elder care. In the wake of global economic disturbances and the weakening ability of governments to sustain social security, many Western states are also exploring the role of the family and filial beliefs and norms in supporting older adults (e.g., Lowenstein and Daatland, 2006; Silverstein et al., 2012). We provided some examples of how researchers can use the DFPM to integrate various concepts proposed by western scholars regarding intergenerational interaction with parents during specific developmental stages or within specific contexts to investigate issues relevant to the parent–child relationship and elder care. We hope that contextualized analysis of issues in Chinese societies and insights from relevant empirical findings based on the DFPM may stimulate research, practical applications, and policy development in other aging societies.

## Author Contributions

OB and KY substantial contributions to the conception or design of the work and analysis and interpretation of concepts, revising it critically for important intellectual content, final approval of the version to be published, agreement to be accountable for all aspects of the work in ensuring that questions related to the accuracy or integrity of any part of the work are appropriately investigated and resolved.

## Conflict of Interest Statement

The authors declare that the research was conducted in the absence of any commercial or financial relationships that could be construed as a potential conflict of interest.
